# Fingerprint of silicic magma degassing visualised through chlorine microscopy

**DOI:** 10.1038/s41598-018-37374-0

**Published:** 2019-01-28

**Authors:** Shumpei Yoshimura, Takeshi Kuritani, Akiko Matsumoto, Mitsuhiro Nakagawa

**Affiliations:** Department of Earth and Planetary Sciences, Hokkaido University, Sapporo, 060-0810 Japan

## Abstract

Volatile-rich silicic magma erupts either explosively as a jet of a mixture of pyroclasts and high-temperature gas, or non-explosively to effuse lava. The bifurcation of the eruption style is widely recognised as being controlled by the efficiency of open-system gas loss from vesiculated magma during ascent. However, the fundamental question of how the gas escapes from highly viscous magma still remains unsolved because the pathways of gas flow are rarely preserved in dense lava. Here we show that such pathways are visualised in groundmass glass using high-resolution chlorine (Cl) mapping analysis on the rhyolitic lava of the Mukaiyama volcano, Japan. The results showed that the glass was highly heterogeneous in Cl content. A spatial distribution of the Cl content in the groundmass glass showed that volatiles diffused towards most bubbles, but the bubbles collapsed into the dense melt rather than growing. All observations, in combination with melt inclusion analysis, indicate that vesiculation, the formation of interconnected bubble channels, open-system gas loss via the channels, and channel collapse repeated within the period of a few days to two weeks during ascent. This cycle repeated individually in centimetre-sized portions of magma with different timing.

## Introduction

Silicic magma eruption has threatened human life as it frequently devastated towns by generating pyroclastic flows or wide-spreading volcanic ash and pumice. Such explosive eruptions also exert a significant influence on climate change, as observed after the 1991 Pinatubo eruption^1^. However, the same silicic magma can also erupt in an entirely different manner, effusing lava non-explosively which causes very limited damage. The style of eruption is controlled by two competing processes: “vesiculation” and “open-system gas loss”^2–5^. Vesiculation is a process in which volatiles that are originally dissolved in the silicate melt at depth exsolve as separated gas bubbles due to a decrease in the pressure-dependent solubility when the magma ascends. The resulting bubbles then expand as a result of decompression, causing further magma ascent because of the enhanced buoyancy. Therefore, vesiculation constitutes a primary driving force for eruption. On the other hand, open-system gas loss is a process in which the exsolved gas escapes from the vesiculated magma, reducing the eruption driving force. Therefore, the efficiency of an open-system gas loss is key to controlling eruption style. If an open-system gas loss dominates, then magma erupts non-explosively, discharging gas-poor dense lava. On the contrary, if open-system gas loss is less effective, then magma erupts vigorously, discharging a mixture of gas and bubbly magma fragments.

The mechanism of an open-system gas loss has long been unclear, because no direct evidence for this process was preserved in dense lava samples. Buoyant migration of individual bubbles, as seen in fizzy drinks, is ineffective because silicic magma has extremely high viscosity (typically >10^7^ Pa s). A long-standing model is that bubbles interconnect to produce permeable channels and gas flows via these^6,7^. Although this model has been the cornerstone of eruption models, it still remains hypothetical because lava rarely preserves bubble channels. A more recent model is gas flow via shear-induced fracture networks^8–13^. This process may play a limited role, however, because fractures form too sparsely to release most of the gas from the magma batch^14^. Here, we show direct evidence that repetition of vesiculation, bubble interconnection, and gas loss through the bubble channels are responsible for the open-system gas loss based on high-resolution Cl mapping analysis of a glassy lava.

We had a unique opportunity at Mukaiyama volcano in Niijima Island, Japan, to study how open-system gas loss proceeds, as a dome-forming lava from this volcano exhibited the transitional state from vesicular, bubbly magma to bubble-free, obsidian-like magma. This transitional state may have remained due to the extremely low temperature of the Mukaiyama magma (ca. 750 °C, estimated using a magnetite-ilmenite geothermometer^15^), which lowers the volatile diffusivity and increases the melt viscosity. In A.D. 886, Mukaiyama first explosively erupted to generate a violent volcanic surge, depositing tephra of a maximum thickness of ~100 m extending over a few kilometres^16^. Activity then shifted to the formation of several pyroclastic cones. Finally, a large, glassy lava dome emerged and activity ceased. Throughout the activity, biotite-rhyolite magma erupted and the chemical composition did not change (Table 1). Based on the phase diagram^17^, the water-saturation pressure of pre-eruptive Mukaiyama magma was estimated to be ~100 MPa, which corresponds to a depth of ~5 km. The initial water content was calculated^18^ as ~4.4 wt%. We investigated the degassing processes based on texture, chlorine distribution, and water content of three representative lava samples (Samples A, B, and C) collected at a working quarry on the Mukaiyama lava dome.

**Table 1:** Groundmass glass composition, water content, microlite crystalinity, and vesicularity of Mukaiyama lava (Samples A–C) and pumice.

Glass composition, wt% (1σ)	Lava sample A	Lava sample B	Lava sample C (obsidian-like part)	Lava sample C (vesicular part)	Pumice from explosive eruption
SiO_2_	76.75 (0.21)	76.60 (0.26)	77.32 (0.19)	77.32 (0.15)	77.31 (0.39)
TiO_2_	0.05 (0.02)	0.06 (0.03)	0.06 (0.02)	0.07 (0.02)	0.06 (0.01)
Al_2_O_3_	12.32 (0.08)	12.29 (0.09)	12.42 (0.04)	12.42 (0.08)	12.16 (0.07)
FeO^t a^	0.64 (0.06)	0.62 (0.05)	0.65 (0.06)	0.63 (0.04)	0.66 (0.03)
MnO	0.07 (0.02)	0.07 (0.02)	0.08 (0.02)	0.07 (0.01)	0.06 (0.01)
MgO	0.08 (0.01)	0.07 (0.01)	0.07 (0.01)	0.07 (0.01)	0.08 (0.01)
CaO	0.46 (0.01)	0.38 (0.01)	0.39 (0.01)	0.40 (0.01)	0.47 (0.01)
Na_2_O	4.43 (0.07)	4.34 (0.08)	4.51 (0.06)	4.47 (0.09)	4.44 (0.06)
K_2_O	3.90 (0.03)	4.07 (0.04)	4.12 (0.04)	4.09 (0.04)	3.84 (0.04)
Cl	0.09 (0.01)	0.08 (0.01)	0.08 (0.01)	0.08 (0.01)	0.09 (0.01)
S	BDL	BDL	BDL	BDL	BDL
Total	98.78	98.58	99.70	99.62	99.17
Groundmass water content^b^ (wt%)	0.06 (0.01)	0.17 (0.01)	0.37−0.47^c^	0.23	n.d.
Microlite crystalinity^d^ (area%^e^)	0.04	0.57	0.23	0.15	n.d.
Vesicularity (area%^e^ or vol%^f^)	56^f^	48^f^	0	24^e^	61^f^
Water-saturation pressure^g^ (MPa)	0.03 (atmospheric)	0.23	1.08−1.72	0.42	n.d.

^a^Total FeO.

^b^Measured with FT-IR.

^c^Water content varied with position (See Fig. 4).

^d^Microlites were plagioclase and pyroxene.

^e^Measured with image analysis of backscattered electron images .

^f^Calculated from volume and mass of a rectangular shaped sample and melt density of 2300 kg/m^3^.

^g^Calculated based on groundmass water content and solubility law of ref.^35^.

## Results and Discussion

**Figure 1:**
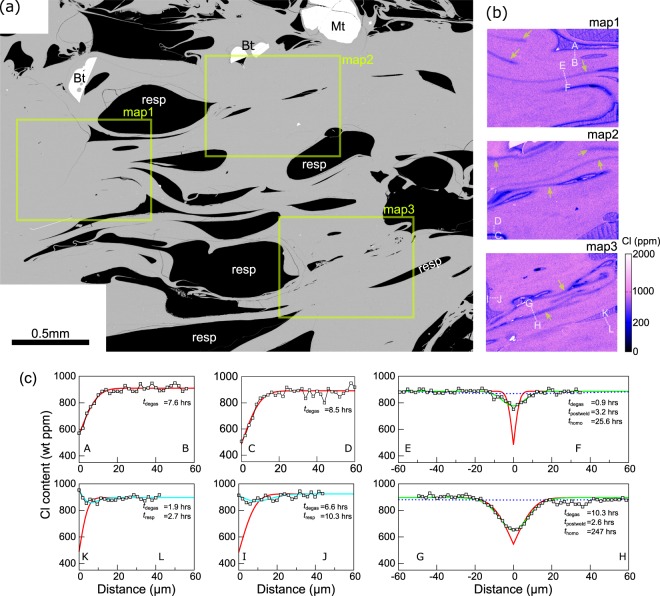
Microstructure and chlorine distribution in the Mukaiyama lava (Sample A). (**a**) A representative back-scattered electron image of Mukaiyama lava. Black denotes a bubble, and grey denotes groundmass glass. Biotite (Bt) and magnetite (Mt) phenocrysts are shown in white. Bubbles deformed and elongated along basically one direction (the lateral direction in this picture). (**b**) The Cl-content maps of rectangular areas in (**a**). For most of the bubbles, the Cl content decreases towards the bubble–melt interface, indicating that diffusive degassing is occurring. A low-Cl tail extends from the acute, pointed edges to far afield or neighbouring bubbles (arrows). For some bubbles (marked as “resp” in (**a**)), the Cl content increases towards the interface, indicating that these bubbles are resorbing into the melt. (**c**) The Cl-content profiles along the white dotted lines in (**b**). Traverses A–B and C–D represent diffusive-degassing profiles towards open bubbles. Traverses E–F and G–H show the U-shaped profile across the low-Cl tail. Traverses I–J and K–L represent the bubble-resorption profile. Red curves indicate the best-fit profile for the diffusive degassing. Green indicates the best-fit profiles for diffusive homogenisation after the bubble-melt interface was welded into a coherent melt. Blue dotted lines indicate the nearly completely homogenised profiles. Cyan indicates the backward diffusion profiles for resorbing bubbles. The calculation method is provided in the Methods.

A backscattered electron image of a Mukaiyama lava sample (Sample A) showed that bubbles elongated basically along one direction (Fig. 1a and Supplementary Fig. S1). Melt films between the bubbles often thin out or fold into a curtain shape, indicating that the magma underwent significant deformation. Bubbles exhibit a complex outline composed of multiple curvatures, rather than a single ellipsoid, suggesting that bubble coalescence and deformation were common. It is also remarkable that many bubbles have an acute, pointed edge at one or more ends (Fig. 1a). Such acute edges suggest that bubbles were being pinched off under compaction and deformation. The water content of the groundmass glass was found to be 0.06 (±0.01) wt% by FT-IR analysis (Table 1). The glass water content was spatially uniform with a spatial resolution of 30–50 μm. This water content indicated that Sample A had degassed almost completely under atmospheric pressure. No dissolved CO_2_ was detected in the IR spectrometry. The vesicularity was 56 vol%, which is much lower than expected under an assumption that closed-system vesiculation occurred: If the initial water (4.4 wt%) exsolved as gas but remained in the magma, then the magma vesicularity would be >99% under atmospheric pressure. Therefore, the observed lava must have lost >90% of its exsolved gas during magma ascent as a result of open-system gas loss.

To investigate how the open-system gas loss occurred, we applied high-resolution Cl mapping analysis to the groundmass glass of the Mukaiyama lava using a field-emission electron probe microanalyser (FE-EPMA) (Methods). Cl is a unique tracer to track degassing processes, because Cl diffusivity is much lower than that of H_2_O and other volatiles^19^, allowing it to potentially preserve degassing-related heterogeneity. The Cl mapping analysis also has a significant advantage in that it allows high spatial-resolution analysis (<2 μm), which is essential to detect and characterise fine-scale Cl heterogeneities presented as follows. Although some studies analysed diffusive heterogeneity of H_2_O and CO_2_ in obsidian to discuss degassing dynamics^20–23^, this method could not be applied to our samples because the Mukaiyama lava was extremely deficient in H_2_O and free of CO_2_.

**Figure 2:**
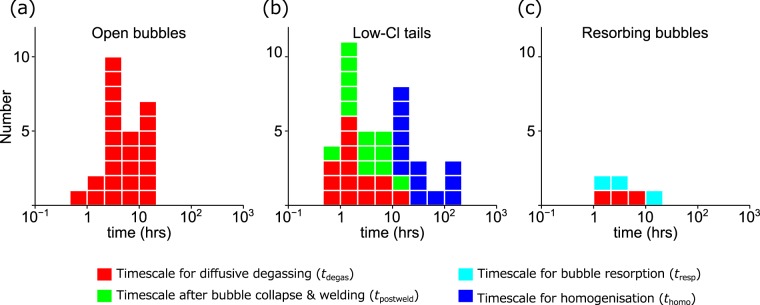
Timescale for degassing processes estimated based on Cl diffusion analysis. (**a**) The timescale of the diffusive degassing for open bubbles (*t*_degas_). This timescale represents the “age” of the bubble. (**b**) The timescales of initial diffusive degassing (*t*_degas_, red), the timescale after collapse (*t*_postweld_, green), and the time required to nearly homogenise the U-shaped profile (*t*_homo_, blue) for the low-Cl tails. The sum of the timescale of diffusive degassing and that after collapse (*t*_degas_ + *t*_postweld_) represents the age. (**c**) The timescales of initial diffusive degassing (*t*_degas_, red) and subsequent resorption (*t*_resp_, cyan) for resorbing bubbles. The sum of these timescales (*t*_degas_ + *t*_resp_) represents the age of this bubble. It is notable that the age is similar among all types of the bubbles, indicating that the timing of vesiculation was nearly the same (a few hours to a day before quenching of lava). The calculation method is provided in the Methods.

The Cl-content maps of Sample A (Fig. 1b and Supplementary Fig. S1) show that various types of heterogeneity exist (The existence of Cl heterogeneity in lavas has previously been reported for other volcanoes^24^). For most bubbles, the Cl content decreased towards the bubble-melt interface (Fig. 1c, Profiles A–B and C–D, and Supplementary Figs S1 and S2), indicating that diffusive degassing was occurring. The timescale of Cl diffusive degassing (*t*_degas_) was calculated by fitting a semi-infinite diffusion equation to the analysed data (Methods), and *t*_degas_ was 0.96–20.7 hours (Fig. 2a, All numerical values are provided in Supplementary Table S1). In addition, many of these bubbles had a long, low-Cl tail extending from an acute edge to far afield or a neighbouring bubble (Fig. 1b, arrows). Based on these observations, the tail is interpreted to represent the collapsed part of the bubble; the bubble-melt interfaces of the collapsed part were then welded into a coherent melt. The tail length was from ~100 μm to >1 mm, indicating that it had been an elongated bubble channel before it collapsed. The Cl-content traverse across the tail shows a U-shaped shallow profile, rather than a V-shaped deep profile (Fig. 1c Profiles E–F and G–H, and Supplementary Figs S1 and S2). This indicates that the tail is being dissipated via diffusive homogenisation. To summarise these observations, diffusive degassing occurred towards bubbles, but the bubbles collapsed into the dense melt instead of growing. This fact clearly indicates that the exsolved gas escaped to the outside of the magma via interconnected bubble channels. Here, the open-system nature is important: if a closed-system were maintained, then bubbles would grow rather than collapse. In the case of already-collapsed bubbles (low-Cl tails), the timescale of initial diffusive degassing (*t*_degas_) and the timescale after bubble collapse and welding (*t*_postweld_) were calculated by fitting the diffusion-welding model equation to the U-shaped profiles (Methods). The calculation result showed that *t*_degas_ was 0.53–10.3 hours and *t*_postweld_ was 0.47–12.8 hours (Fig. 2b, Supplementary Table S1). The timescale after formation of the bubbles (*t*_degas_ + *t*_postweld_) was 4–14 hours, within the same range of timescale of other open bubbles.

It was also observed that, for some bubbles, the Cl content increased towards the bubble-melt interface (Fig. 1c, Profiles I–J and K–L, and Supplementary Figs S1 and S2). Such Cl distribution is produced when the Cl chemical potential of a gas exceeds that of the surrounding melt. Bubble resorption is the most likely process for this, as the gas becomes extremely Cl-rich upon resorption because Cl diffusion is much slower than that of water and other volatiles, namely, diffusive fractionation occurs^25^. The possible causes for the resorption include magma repressurisation and progressive melt dehydration. Repressurisation may occur when lava undergoes a conveyor belt motion^26^. Progressive melt dehydration is also a common process, because the water content of melt decreases endlessly as long as an open gas channel is maintained^27^. Another possibility could be lava cooling, which may induce bubble resorption due to the increase in volatile solubility^28^. This latter process is, however, unlikely in the present case, because if the cooling were responsible for the resorption, then all bubbles would have a Cl-rich area at the bubble-melt interface. The timescale for the initial diffusive degassing (*t*_degas_) and that for subsequent resorption (*t*_resp_) were calculated by fitting a diffusion-resorption model to the observed profiles (Methods). The calculation result showed that *t*_degas_ was 1.9–9.4 hours and *t*_resp_ was 2.7–10.3 hours (Fig. 2c, Supplementary Table S1). The total timescale (*t*_degas_ + *t*_resp_) was 4.6–16.9 hours, similar to the timescale for other open bubbles and already-collapsed bubbles (low-Cl tail). We suggest that all bubbles formed at a similar time (a few hours to a day before quenching of lava), but some of them were left isolated. The isolated bubbles then resorbed into the melt.

Melt inclusion analysis provides further insight into how the degassing processes proceeded in the ascending magma. Cl content of melt inclusions in quartz and plagioclase phenocrysts from explosive pumice, which may represent the initial chlorine content in the magma chamber, was 1200 ± 300 ppm (n = 12), with the highest value being 2100 ppm (Supplementary Table S2). The average value of 1200 ± 300 ppm is significantly higher than the Cl content of the least-degassed, non-vesiculated part of the groundmass (~900 ppm; Fig. 1c). This observation indicates that even the least-degassed part of the groundmass had undergone vesiculation, open-system gas loss, and compaction before the last degassing event initiated. The fact that the least-degassed part is nearly homogeneous in the Cl content indicates that the degassing-related heterogeneity, such as U-shaped profiles, produced by the preceding degassing event had been erased, due to the diffusive homogenisation before the last event initiated. Thus, we calculated the timescale required to homogenise the U-shaped profiles (*t*_homo_) (Methods), which was found to be 10.9–323 hours (Fig. 2b, Supplementary Table S1). This indicates the two degassing events occurred with an interval of a half day to two weeks.

**Figure 3:**
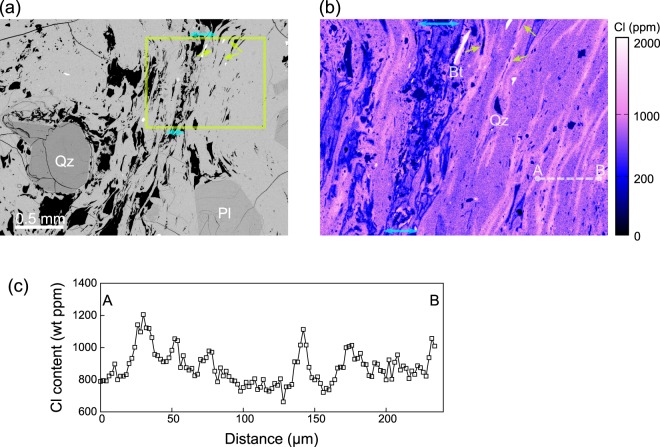
A lava sample that may represent the deeper part of the Mukaiyama lava dome (Sample B). (**a**) The backscattered electron image; the bubbles align along one direction (the subvertical direction in this image). The bubbles have an irregular shape because of intensive deformation. Qz and Pl represent quartz and plagioclase phenocrysts, respectively. (**b**) The Cl-content map of the rectangular area in (**a**). High-Cl stripes run along the direction of bubble elongation. Arrows indicate small deformed bubbles in the high-Cl stripe, which are in the process of being resorbed into the melt. A vesicular, apparently permeable path is observed near the high-Cl stripes (the area between the cyan double-headed arrows), allowing continuous dehydration. Bt represents biotite phenocryst. (**c**) Shows the Cl-content profile along the line A–B, as shown in (**b**). Multiple peaks with high Cl contents represent the high-Cl stripes.

Similar degassing events might have occurred at multiple depths, as suggested by an additional lava sample (Sample B). Sample B has higher water content and lower vesicularity compared to Sample A (Table 1), indicating that it was emplaced at a deeper part of the lava dome (0.23 MPa). Note however that this emplacement pressure is the lower limit, because the lava is highly vesicular and might have undergone significant open-system gas loss, which decreases the water content to below the water-saturation level. The groundmass glass preserved a highly heterogeneous Cl distribution—high-Cl stripes run along the direction of bubble elongation (Fig. 3). Several small, deformed bubbles are observed in the high-Cl stripes (arrows in Fig. 3), suggesting that these bubbles are in the process of being resorbed into the melt. The high-Cl stripes thus represent the remnant of the elongated bubble-melt interface of resorbed bubbles. The most likely cause for the resorption is progressive melt dehydration. A vesicular, apparently permeable path is observed near the stripes (the area between upper and lower double-headed arrows in Fig. 3a,b), allowing continuous dehydration. The Cl content near this path is much lower than that within the stripes, which is consistent with the open-system nature. The possibility that Sample B is the repressurised product of a shallow-emplaced sample such as Sample A can be excluded, because Sample A is so deficient in H_2_O that it cannot produce Sample B in terms of vesicularity and glass water content. In summary, vesiculation, open-system gas loss, bubble resorption, and compaction might have occurred in the deeper part.

The fact that the degassing event occurred over a few hours to a day while the interval between the events ranged from a half day to two weeks, in combination with the fact that the degassing occurred at various depths, indicates that the magma repeatedly underwent two-step processes, with an initial few hours to a day of vesiculation, open-system degassing, bubble channel collapse, and bubble resorption, followed by an interval of a half day to two weeks. One possible explanation for such repetitive two-step processes (cyclic degassing) is the competition between kinetically limited vesiculation and subsequent bubble elimination: During ascent, the degree of volatile supersaturation increases. When it overcomes the kinetic barrier, magma vigorously vesiculates. The vesiculated magma then transitions into bubble-free magma via open-system gas loss and resorption. The next vesiculation occurs when the magma again increases the degree of supersaturation. If the open-system gas loss causes water undersaturation, then it may take longer to increase the degree of supersaturation for the subsequent vesiculation. One may consider another possibility that magma repeatedly underwent ascent and stagnation to cause the cyclic degassing. However, this scenario is incompatible with the asynchronous, spatially-heterogeneous nature of the degassing, as explained in the following paragraph.

**Figure 4:**
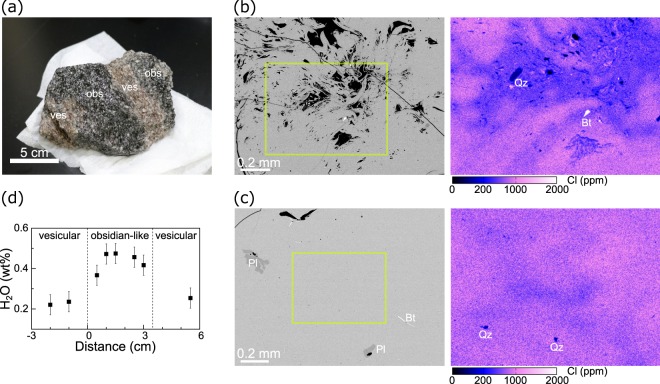
A lava sample containing both vesicular and obsidian-like regions in a single block (Sample C). In (**a**), the darker part (“obs”) represents the obsidian-like region, while the light-coloured part (“ves”) represents a bubble-rich vesiculated region. Both regions have a thickness of a few centimetres. (**b**) The back-scattered electron image and the Cl-content map of the vesicular region. The groundmass glass is highly heterogeneous in terms of Cl content, indicating that vesiculation, open-system gas loss, and compaction occurred in this region. Qz and Bt represents quartz and biotite phenocrysts, respectively. (**c**) The back-scattered electron image and the Cl-content map of the obsidian-like region. The Cl content is more homogeneous compared to that of (**b**), indicating that this region was previously vesiculated, but later transitioned into its current state. Pl represents plagioclase phenocrysts. (**d**) The water content of the groundmass glass across the obsidian-like and vesicular regions.

Cyclic degassing may have occurred in each small portion of magma with a different timing and period, rather than synchronously throughout the entire magma. This idea was obtained from an additional lava sample (Sample C) that contained both a bubble-rich, vesicular region and a bubble-free, obsidian-like region in a single block (Fig. 4a). The bubble-rich region exhibited highly heterogeneous Cl distribution, similar to the Sample A and B, indicating that this region preserves on-going degassing and compaction processes (Fig. 4b). In contrast, the obsidian-like region was nearly bubble-free, and the Cl distribution was more homogeneous, although marble-like patterns weakly remained (Fig. 4c). This indicates that the obsidian-like region had been vesicular, but later transitioned into a bubble-free melt. The water content of the obsidian-like region was ~0.47 wt% (Fig. 4d), indicating that the last transition event occurred at a depth of ~80 m. On the other hand, the bubble-rich region had a lower water content (0.23 wt%), suggesting that it did not collapse at that depth (or at the corresponding time) and the open-system gas loss continued. The fact that the two contrasting regions coexist within a few centimetres, and that the timing of the degassing is different in each region, indicates that the cyclic degassing occurred heterogeneously and non-synchronously on both spatial and temporal scales. This view is supported by the observation that the interior of one of older rhyolitic lavas on Niijima Island shows a patchy structure composed of an obsidian-like region and vesicular region (Supplementary Fig. S3).

## Conclusion

**Figure 5:**
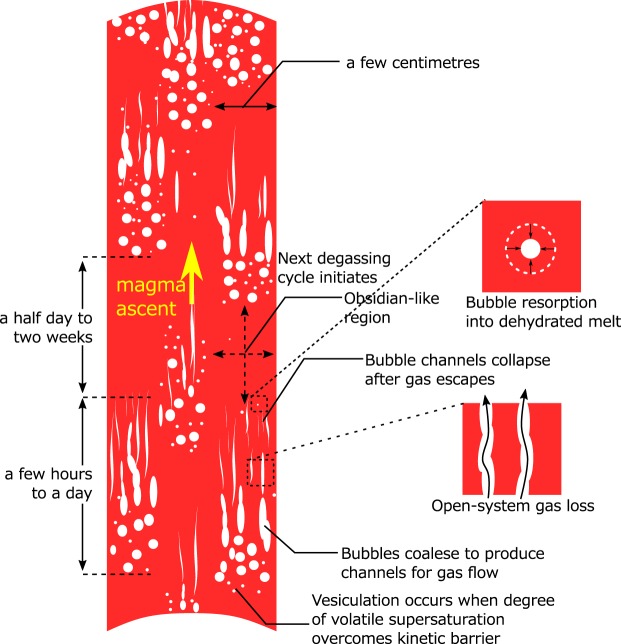
Model of the open-system gas loss during ascent. As the water-rich magma ascends and pressure decreases, the magma vesiculates into a bubbly foam. Continuous magma ascent induces significant deformation, causing bubble interconnection. As the gas permeability increases sufficiently, the gas flows to the outside of the magma. The bubble channel then collapses into the coherent melt, and the magma transitions into a bubble-less, dense lava. Isolated bubbles may resorb into the dehydrated melt, leaving a nearly bubble-free melt. The time required for the aforementioned processes is a few hours to a day. After the transition, the magma does not vesiculate until the degree of volatile supersaturation overcomes the kinetic barrier for the next vesiculation. Meanwhile, the Cl heterogeneity is nearly erased because of diffusive homogenisation. This duration is a half day to two weeks. When the degree increases sufficiently, the magma again initiates another cycle of vesiculation, bubble-channel formation, open-system gas loss, and magma compaction. The cycle may be repeated several times before magma emerges. This cyclic degassing occurs in few-centimetre-sized portions of magma with a different timing and period of degassing, rather than synchronously throughout the whole magma batch.

We conclude that the following could be the primary mechanism for open-system gas loss at shallow levels (Fig. 5). As a volatile-rich magma ascends, it vesiculates into bubbly magma. Bubbles then coalesce to produce interconnected bubble channels under strong shear deformation during flow^29^. After the gas escapes to the outside via the bubble channel, the channel collapses and is welded into coherent melt. Isolated bubbles resorb into the dehydrated melt, leaving bubble-free magma. These processes took a few hours to a day. Then, a half-day to two-week period during which volatile supersaturation builds up for the next vesiculation follows. These two-step processes are repeated several times in each portion of magma. The timing of the cycle is different in each portion. The depth at which each degassing cycle occurred is not well constrained, because the groundmass water content of highly vesicular lava might have decreased to below the water-saturation level when it underwent the open-system gas loss. Whether cyclic degassing also occurs at depth remains to be shown.

This degassing pattern was fortunately preserved because the efficiency of the degassing was low in the low-temperature Mukaiyama magma. If the degassing cycles had proceeded more efficiently, then the transition into a bubble-free lava would be more rapid, producing a nearly completely bubble-free obsidian flow. In contrast, if the efficiency were too low, then a violent explosive eruption would result. We thus suggest that the eruption styles, from lava effusion to a violent explosion, are continuous, being modulated by the efficiency of the cyclic degassing. The ratio of the vesicular part to the obsidian part of a volcanic rock may be a useful measure of the efficiency of cyclic degassing.

Our degassing model may also explain the recently recognised hybrid eruption in the Chaitén and Cordón Caulle volcanoes, in which lava effusion occurred simultaneously to explosive eruption^30^. For hybrid eruption, a degassing model involving repetition of closed-system exsolution and ensuring pulses of vapour extraction through fracture networks was proposed^30^. However, uncertainty remains about the efficiency of degassing because fractures formed too sparsely (spacing of tuffisite veins is typically more than a few centimetres^31^). Our model may overcome this difficulty, because it involves 1) pervasively produced bubble channels with a much closer channel spacing of ~100 μm, which is the distance between open bubbles or low-Cl tails and, 2) repeated closed and open-system degassing steps.

## Methods

### Chlorine mapping analysis

Cl-content mapping analysis was conducted using a JEOL JXA-8530F field-emission electron probe microanalyser (FE-EPMA) installed at Hokkaido University. For beam calibration, natural and synthetic crystals were used as follows: SiO_2_ for Si, TiO_2_ for Ti, Al_2_O_3_ for Al, Fe_2_O_3_ for Fe, MnO for Mn, MgO for Mg, wollastonite for Ca, jadeite for Na, K-feldspar for K, halite for Cl, and BaSO_4_ for S. The Cl count was measured using a PETH crystal, which enabled us to obtain higher count rates (~330%) compared to those of a traditional PET crystal. The acceleration voltage and beam current for mapping analysis was 15 kV and 100 nA, respectively. The high beam current is important to obtain a higher count rate for Cl. However, because of the severe beam damage effect, nearly all the Na was lost during the mapping analysis. The beam diameter was 2 μm. The mapping analysis was conducted by moving the stage by 2 μm along the *x* and *y* directions for each step. Dwell time at each step was 500 ms. The map size was typically 0.84 mm × 0.63 mm (420 steps × 315 steps). It took ca. 19 h to acquire a map. Following the mapping analysis, the Cl count rate at each point was converted to absolute Cl content based on a calibration line, which was established for each map by comparing the Cl count rate at several points to the absolute Cl content at the same points. The absolute Cl content was determined via standard quantitative analysis; the acceleration voltage and beam current were 15 kV and 10 nA, respectively. A ZAF correction method was applied to all analyses. The beam diameter was 2 μm.

### Water-content analysis

The water content of groundmass glass was analysed using a JASCO FT/IR-6600 equipped with an IRT-5200VC installed at Hokkaido University. A doubly polished glass wafer was prepared and analysed using a transmission mode. The beam aperture was 30 μm × 30 μm. A mercury-cadmium-telluride (MCT) detector was used to obtain spectra. The total water content (H_2_O molecules + OH) was quantified based on the Beer–Lambert law and the absorbance of the peak at 3550 cm^−1^ that represents the X-OH stretching vibration. A molar absorption coefficient of 75 L mol^−1^ cm^−1^ (ref.^32^) and constant glass density of 2320 kg/m^3^ were used to calculate the total water content. Repeated analysis on the same spot showed that the error (1 σ) was <0.01 wt%.

### Diffusion analysis for open bubbles

For analysis of Cl diffusion profiles around open bubbles, we used a one-dimensional semi-infinite diffusion equation as follows^33^:

1 $$C={C}_{boundary}+({C}_{initial}-{C}_{boundary})\,{\rm{erf}}\,(\frac{x}{2\sqrt{Dt}})$$

where *C* is concentration, *D* is Cl diffusivity, *t* is time, and *x* is distance from the bubble–melt interface. Subscripts *initial* and *boundary* represent the initial and boundary (bubble–melt interface), respectively. The timescale of diffusive degassing (*t*_degas_) was determined by fitting Eq. (1) to the observed profile. *D* was assumed to be 10^−15^ m^2^/s, which is an estimated value for rhyolite with ca. 1 wt% dissolved H_2_O at 750 °C (ref.^19^). *C*_initial_ and *C*_boundary_ were simultaneously determined in this calculation. One-dimensional diffusion was used, rather than spherical coordinate diffusion, because bubbles strongly deformed to an elongated or irregular shape. We did not consider the effect of growth and shrinkage (moving boundary), because the rate and history of these processes are difficult to evaluate. Because Cl diffusivity is strongly dependent on the H_2_O content of melt, and because the H_2_O content continuously decreases during magma ascent, the diffusion timescale may change by <1–2 orders of magnitude.

### Diffusion analysis for low-Cl tails

We assumed that the U-shaped profile across the low-Cl tail is the product of the initial diffusive degassing towards bubble at 0 ≤ *t* ≤ *t*_degas_ and subsequent diffusive homogenisation at *t*_degas_ ≤ *t* ≤ (*t*_degas_ + *t*_postweld_). Here, *t*_degas_ is the timescale of initial diffusive degassing and *t*_postweld_ is the timescale for homogenisation following welding. Channel collapse and welding are assumed to have occurred at *t* = *t*_degas_. The calculation method for this case is identical to that in ref.^34^. At 0 ≤ *t* ≤ *t*_degas_, the one-dimensional diffusion equation,

2 $$\frac{\partial C}{\partial t}=D\frac{{\partial }^{2}C}{\partial {x}^{2}},$$

was solved numerically under an initial condition of *C* (*t* = 0) = *C*_initial_ and the boundary condition of *C* (*x* = 0) = *C*_boundary_. This produces a simple decreasing profile towards the bubble, identical to the aforementioned case (Eq. (1)). For *C*_initial_, we used the average of Cl contents at regions far afield from the U shape. *C*_boundary_ was assumed to be identical to *C*_boundary_ of open bubbles near the tail. At *t* = *t*_degas_, the boundary condition was removed to simulate disappearance of the bubble-melt interface (bubbles collapse and welding). Following removal of the boundary condition, Eq. (2) was further solved until *t* = *t*_degas_ + *t*_postweld_. The resultant profile has a mildly curved shape. If this profile is symmetrically juxtaposed at *x* = 0, then the complete traverse has a shallow, U-shaped profile. *t*_degas_ and *t*_postweld_ were determined such that the resultant U-shaped profile best fits the observed profile. The time required to nearly completely homogenise the U-shaped profile (*t*_homo_) was calculated by continuing the above diffusion calculation until the Cl content at the bottom of the U-shaped profile (*x* = 0) reached 95% of the initial Cl content (*C*_initial_). The value of 95% was chosen because far-afield Cl contents often fluctuates within <10% in the observed profiles.

### Diffusion analysis for resorbing bubbles

For the resorbing bubbles, it was assumed that diffusive degassing occurred at 0 ≤ *t* ≤ *t*_degas_, and backward diffusion (resorption) followed at *t*_degas_ ≤ *t* ≤ (*t*_degas_ + *t*_resp_). Here, *t*_resp_ represents the timescale of resorption. At 0 ≤ *t* ≤ *t*_degas_, Eq. (2) was solved numerically under an initial condition of *C* (*t* = 0) = *C*_initial_ and the boundary condition of *C* (*x* = 0) = *C*_boundary_. This produces a simple diffusive degassing profile. For *C*_initial_, we used the average of the Cl content at the region far afield from the bubble-melt interface. *C*_boundary_ was assumed to be identical to *C*_boundary_ of bubbles near the tail. At *t* = *t*_degas_, the volatile degassing towards the bubble changed to bubble resorption. This was expressed by a change in the boundary condition to *C* (*x* = 0) = *C*_boundary-high_ (*C*_boundary-high_ > *C*_boundary_), where *C*_boundary-high_ is the new Cl content at the boundary. *C*_boundary-high_ was set to the observed high Cl content at the bubble-melt boundary of the resorbing bubble. Eq. (2) was further solved until *t* = *t*_degas_ + *t*_resp_. *t*_degas_ and *t*_resp_ were determined such that the resultant profile best fits the observed profile.

## Supplementary information

Supplementary Material
