# Supplementary material for: Fingerprint of silicic magma degassing visualised through chlorine microscopy
Source: Sci Rep. 2019 Jan 28;9:786. doi: 10.1038/s41598-018-37374-0 (PMC6349900; doi:10.1038/s41598-018-37374-0)
Supplement: Supplementary file 1 — Supplementary Material [file 41598_2018_37374_MOESM1_ESM.pdf]

Fingerprint of silicic magma degassing visualised through chlorine microscopy

Shumpei Yoshimura, Takeshi Kuritani, Akiko Matsumoto & Mitsuhiro Nakagawa

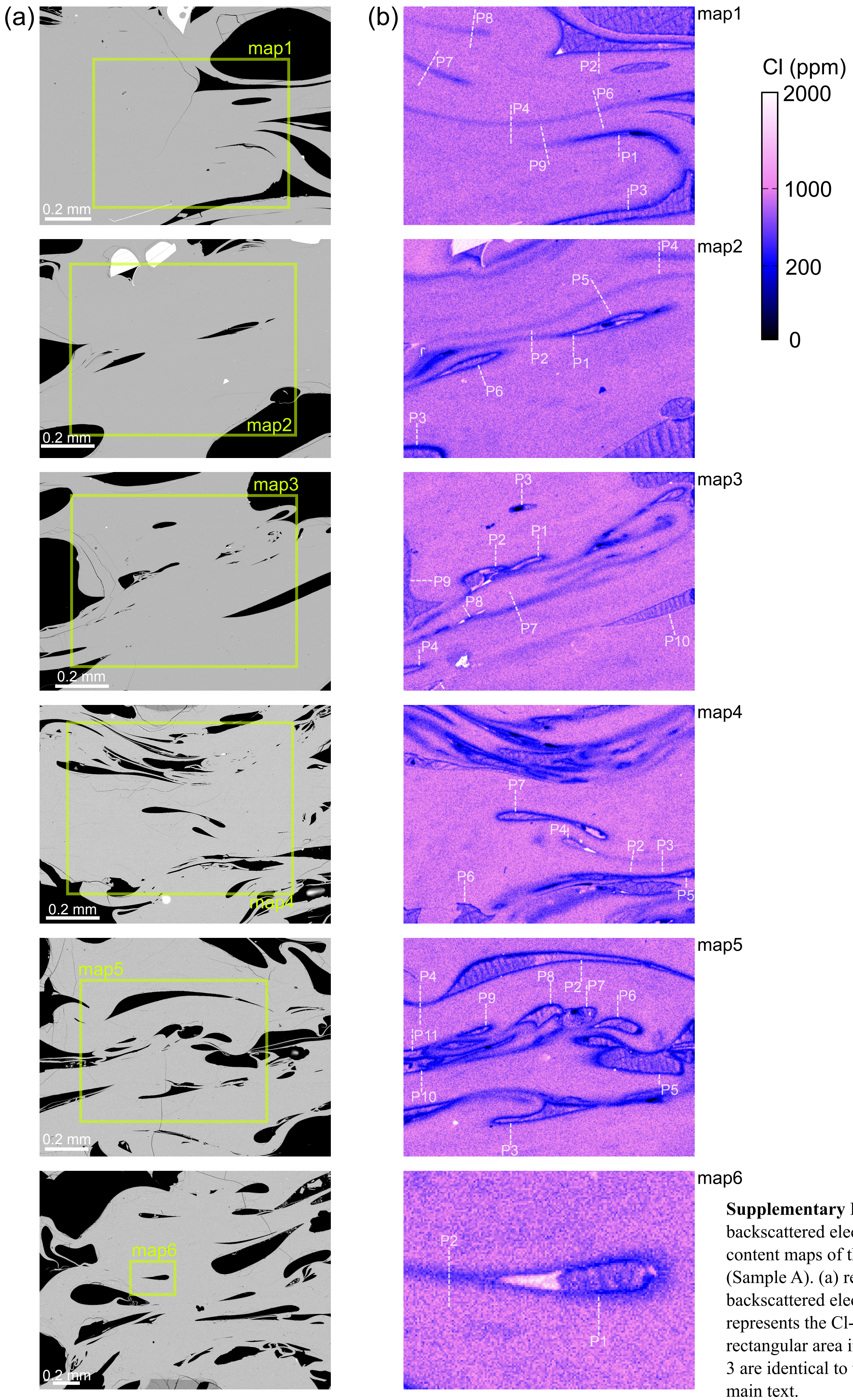

Supplementary Figure S1. Additional backscattered electron images and Cl-content maps of the Mukaiyama lava (Sample A). (a) represents the backscattered electron images and (b) represents the Cl-content maps of the rectangular area in (a). Maps 1, 2, and 3 are identical to those in Fig. 1 in the main text.

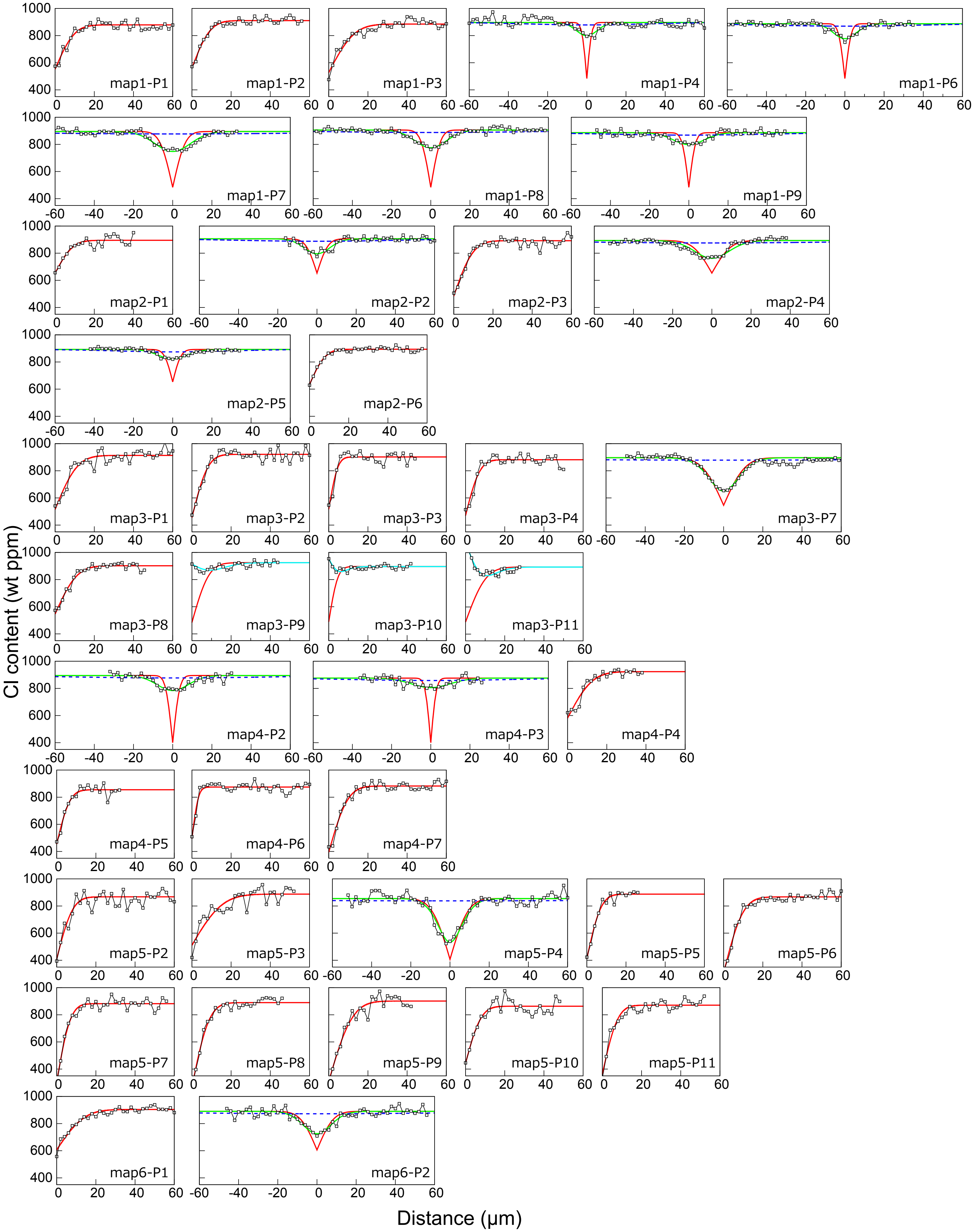

Supplementary Figure S2. Additional Cl-content line profiles of the Mukaiyama lava. The name of the profiles (for example, "map-P1") represents the respective map (map1) and profiles (P1) shown in Supplementary Fig. S1. Profiles A–B, C–D, E–F, G–H, I–J, and K–L in Fig. 1c in the main text are, respectively, identical to map1-P2, map2-P3, map1-P6, map 3-P7, map 3-P9, and map3-10. Red curves indicate the best-fit profile for the diffusive degassing. Green indicates the best-fit profiles for diffusive homogenisation after the bubble–melt interface was welded into a coherent melt. Blue dotted lines indicate the nearly completely homogenised profiles. Cyan indicates the backward diffusion profiles for resorbing bubbles. The calculation method is provided in Methods. The results of all t_{degas}, $t_{postweld}$, t_{homo} and t_{resp} are listed in Supplementary Table S1.

(a)

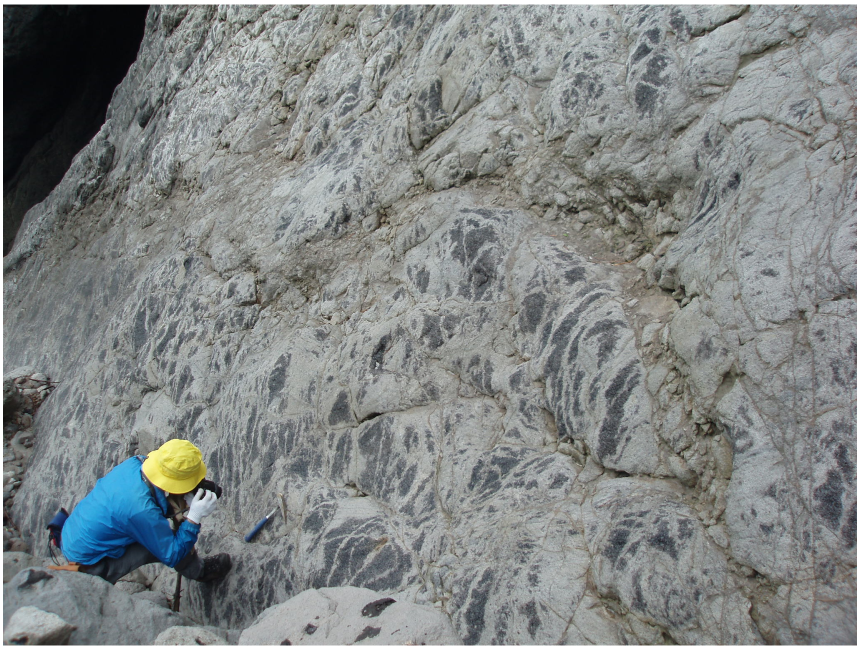

(b)

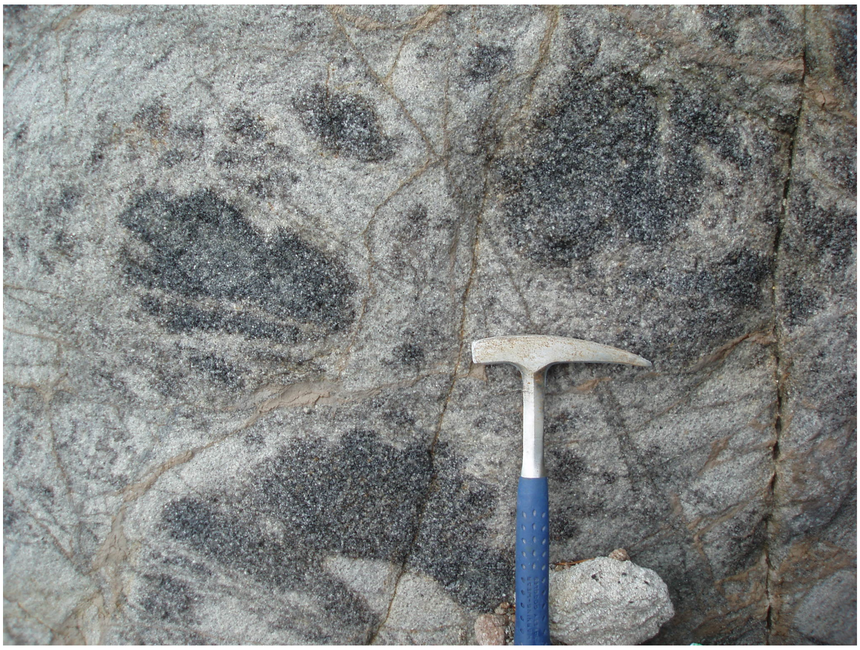

Supplementary Figure S3. Patchy structure composed of a bubble-free, obsidian-like part and a bubble-rich part in one of old lavas on Niijima Island (the Atchiyama lava, ~1600 years old[16]). The black patches are the obsidian-like part, and the white matrix is the vesicular part. (a) shows the outcrop at a steep cliff just above the sea; the lava interior consists of an obsidian-like/ vesicular patchy structure. (b) shows a close-up view of the obsidian/vesicular structure. Photos were taken by Yoshimura.

Supplementary Table S1 Timescales for degassing processes observed in Mukaiyama lava Sample A.

Profile	t_{degas} (h)	$t_{postweld}$ (h)	t_{homo} (h)	t_{resp} (h)
Open bubbles				
map1-P1	8.33			
map1-P2	7.60			
map1-P3	14.46			
map2-P1	7.06			
map2-P3	8.45			
map2-P6	6.13			
map3-P1	11.04			
map3-P2	5.68			
map3-P3	1.82			
map3-P4	4.54			
map3-P8	10.65			
map4-P4	13.51			
map4-P5	3.95			
map4-P6	0.96			
map4-P7	8.12			
map5-P2	5.72			
map5-P3	20.66			
map5-P5	4.57			
map5-P6	8.41			
map5-P7	4.18			
map5-P8	6.71			
map5-P9	12.49			
map5-P10	5.39			
map5-P11	5.25			
map6-P1	16.73			
Already-collapsed bubbles (Low-Cl tails)				
map1-P4	0.53	3.53	14.75	
map1-P6	0.86	3.19	25.58	
map1-P7	3.58	0.47	120.22	
map1-P8	2.11	7.61	65.78	
map1-P9	1.00	8.03	25.19	
map2-P2	2.78	4.14	29.19	
map2-P4	7.97	5.94	82.11	
map2-P5	1.56	6.33	10.86	
map3-P7	10.33	2.64	246.86	
map4-P2	1.08	8.14	45.44	
map4-P3	0.69	12.78	20.61	
map5-P4	7.39	1.67	322.83	
map6-P2	6.08	3.69	93.03	
Resorbing bubbles				
map3-P9	6.61			10.28
map3-P10	1.86			2.72
map3-P11	9.39			5.97

Supplementary Table S2 Chemical composition of melt inclusions from Mukaiyama explosive pumice (wt%)

Melt inclusion	08-mi01	10-mi01	10-mi02	11-mi01	13-mi01	14-mi01	14-mi02	16-mi01	17-mi01	18-mi01	18-mi02	18-mi03
Host phenocryst	Qz	Qz	Qz	Qz	Pl	Pl	Pl	Qz	Qz	Qz	Qz	QZ
SiO_2	71.37	71.36	71.36	72.50	72.64	72.14	77.17	72.30	72.66	72.26	72.32	72.52
TiO_2	0.07	0.05	0.05	0.08	0.05	0.04	0.09	0.03	0.05	0.09	0.13	0.11
Al_2O_3	11.51	11.35	11.35	10.69	11.43	11.49	12.22	11.06	11.43	11.78	11.43	11.66
FeO^t (1)	0.59	0.59	0.59	0.68	0.58	0.60	0.66	0.55	0.55	0.53	0.62	0.54
MnO	0.07	0.03	0.03	0.03	0.03	0.06	0.06	0.06	0.10	0.05	0.06	0.08
MgO	0.08	0.06	0.06	0.10	0.06	0.07	0.07	0.09	0.07	0.07	0.08	0.08
CaO	0.41	0.48	0.48	0.54	0.44	0.46	0.49	0.47	0.40	0.46	0.48	0.47
Na_2O	3.82	3.65	3.65	3.55	3.69	3.91	4.66	3.99	3.91	3.86	3.71	3.66
K_2O	3.62	3.63	3.63	3.42	3.88	3.60	3.58	3.55	3.59	3.82	3.93	3.88
Cl	0.10	0.11	0.11	0.12	0.12	0.12	0.12	0.21	0.11	0.10	0.12	0.12
S	nd	0.01	0.01	0.02	0.03	0.02	0.01	0.04	nd	0.01	nd	nd
Total	91.61	91.30	91.30	91.69	92.91	92.49	99.11	92.30	92.86	93.01	92.87	93.10

(1) Total FeO
